# Development of an HPLC method for the simultaneous determination of azithromycin, clarithromycin, and erythromycin in wastewater

**DOI:** 10.1039/d5ra07172k

**Published:** 2025-12-05

**Authors:** Thi Tu Anh Duong, Truong Xuan Vuong, Thi Thao Ta, Thi To Loan Nguyen, Thi Mai Viet Ngo

**Affiliations:** a Faculty of Chemistry, Thai Nguyen University of Education No. 20 Luong Ngoc Quyen Street Thai Nguyen City 24000 Vietnam; b Faculty of Natural Sciences and Technology, TNU-University of Science Tan Thinh Ward Thai Nguyen City 24000 Vietnam xuanvt@tnus.edu.vn; c Faculty of Chemistry, University of Natural Science, Vietnam National University Hanoi Vietnam

## Abstract

The widespread occurrence of macrolide antibiotics in aquatic environments poses serious ecological and public health concerns, highlighting the need for reliable, accessible, and validated analytical methods. In this study, a rapid and cost-effective high-performance liquid chromatography method with photodiode array detection (HPLC-PDA) was developed and validated for the simultaneous determination of azithromycin (AZM), clarithromycin (CLR), and erythromycin (ERY) in wastewater. Chromatographic separation was achieved on an Agilent Zorbax Eclipse C18 column (250 × 4.6 mm, 5 µm) at 35 °C using a mobile phase of acetonitrile and 10 mM NaH_2_PO_4_ buffer (60 : 40, v/v, pH 6.0), flow rate 0.8 mL min^−1^, and injection volume 10 µL. Retention times were 3.76, 4.91, and 6.23 min for AZM, CLR, and ERY, respectively, with baseline resolution (*R*_s_ > 1.5). The method exhibited excellent linearity over 2–15 µg mL^−1^ for all analytes (*R*^2^ = 0.997–0.999). Limits of detection (LOD) were 5–6 µg L^−1^ and limits of quantification (LOQ) were 17–20 µg L^−1^, meeting AOAC (Association of Official Analytical Chemists) performance standards. Intra- and inter-day precision (%RSD) were below 2.0%, and mean recoveries from spiked wastewater samples ranged from 99.1% to 104.5%. Minor deliberate changes in pH (±0.2) or flow rate (±0.1 mL min^−1^) produced negligible effects (<2% deviation), confirming the robustness of the method. Matrix effects were minimal (<3%), demonstrating that the wastewater matrix did not interfere with analyte detection. Application of the validated method to hospital and livestock wastewater samples revealed macrolide concentrations up to 13.36 mg L^−1^ (CLR), 11.27 mg L^−1^ (ERY), and 7.80 mg L^−1^ (AZM). The developed HPLC-PDA method is therefore suitable for routine environmental monitoring, providing a simple and reliable tool for assessing macrolide antibiotic contamination in wastewater.

## Abstract 2

A simple robust, and cost-effective HPLC-PDA method was developed and validated for simultaneously quantifying azithromycin, clarithromycin, and erythromycin in wastewater, offering reliable performance for routine environmental monitoring.

## Graphical abstract

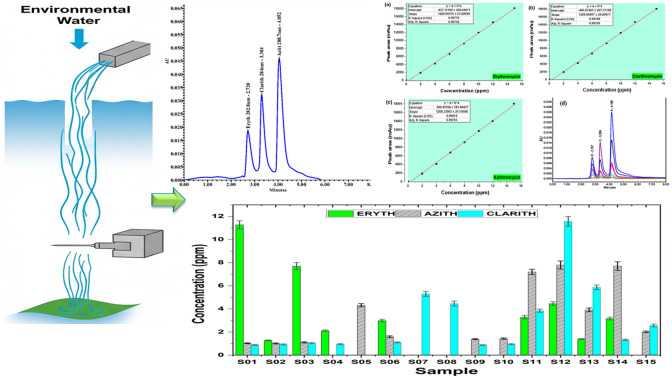

## 1. Introduction

Macrolide antibiotics, including erythromycin and its semi-synthetic derivatives azithromycin and clarithromycin, are used rather widely in human and veterinary medicine for the treatment of many bacterial infections. Macrolide antibiotics are particularly effective for the elimination of a range of pathogens, including intracellular bacteria as well as several opportunistic infectious agents.^1,2^ Macrolides are used most notably in human therapy for conditions such as community-acquired pneumonia and are particularly renowned for their safety in pregnancy and lactation, contributing to their pervasive utilization.^3^ The long-term efficacy and relative safety of these agents further solidify their central role in treatment.

In the veterinary sector, macrolides are also used to prevent and treat bacterial infections in domestic animals, where they have been used a great deal more. This is a trend in the use of antibiotics that not only acts against health concerns of animals but also results in the discharge of antibiotics into the environment, with the potential to bring about the development of antimicrobial resistance (AMR).^4,5^ Research indicates that the extensive application of macrolides in farming can enhance the emergence of resistant bacterial strains, and treatment becomes challenging in the future both in animals and humans.^1,6^ The pressure generated by such antibiotic demands in veterinary practices combined with poor regulatory mechanisms in some regions further worsen the situation.^4,5^

Azithromycin, clarithromycin, and erythromycin are the members of the macrolide group and possess a common structural element: an amino sugar residue and a macrolactone ring. This is responsible for their high lipophilicity and chemical stability, and hence to some extent for their resistance to biodegradation in environmental matrices.^7^ Erythromycin specifically contains a 14-membered lactone ring and is the prototype molecule of this group. Clarithromycin is characterized by the presence of a methoxy substituent, which stabilizes the acid more, while azithromycin, an azalide, contains a 15-membered ring that imparts much greater stability against metabolic degradation.^8^

These structural similarities not only account for their therapeutic efficacy but also explain their prevalence in environmental waters. Studies have detected macrolides in municipal and hospital effluents, commonly in concentrations ranging from tens to thousands of nanograms per liter. This environmental persistence can largely be attributed to incomplete metabolic breakdown in humans and animals, along with the insufficient removal efficiency of conventional wastewater treatment processes.^7^ Such findings illustrate the ecosystem implications of widespread macrolide usage, as these compounds frequently co-occur in aquatic environments, raising concerns about their potential for inducing antibiotic resistance.^8^

The contemporary scholarly literature revealed a spectrum of analytical strategies for macrolide detection that encompass chromatographic and electrochemical techniques, each with unique strengths and limitations. Thin-layer chromatography (TLC) with densitometry has been employed due to its simplicity and cost-effectiveness. Studies have reported TLC-based methods that offer rapid qualitative screening, although these techniques are generally less sensitive compared with more sophisticated systems.^9^ Reversed-phase high-performance liquid chromatography (HPLC) is another well-established approach that can be coupled with diverse detection schemes, including ultraviolet and fluorescence detection, thereby offering robust quantification capabilities while remaining accessible to many laboratories.^9,10^

Additionally, capillary electrophoresis (CE) methods combined with solid-phase extraction (SPE) have been investigated as viable alternatives, particularly for the analysis of trace levels of macrolide residues in complex matrices such as environmental water samples.^11,12^ While CE offers high separation efficiency and low solvent consumption, the necessity for efficient preconcentration *via* SPE can complicate routine implementation in settings with limited resources. In contrast, ultra-high-performance liquid chromatography tandem mass spectrometry (UHPLC-MS/MS) methods have proven exceptionally sensitive and selective. Such techniques, often employing molecularly imprinted solid-phase extraction, provide a powerful means to detect macrolide residues, even at low concentrations, yet they require advanced instrumentation and extensive sample preparation steps.^10,13^

The literature indicates that investigations into the quantification of antibiotics in aquatic systems have largely been limited to single-compound analyses or very small groups of analytes. For instance, while several studies have reported methods employing solid-phase extraction (SPE) coupled with liquid chromatography-tandem mass spectrometry (LC-MS/MS) for the detection of various antibiotics in water matrices,^14,15^ these reports tend to focus on multi-residue methods that typically require advanced instrumentation and elaborate sample preparation steps. Notably, despite the widespread concern over antibiotic residues, there is a lack of published studies that specifically detail the simultaneous determination of azithromycin, clarithromycin, and erythromycin using conventional high-performance liquid chromatography (HPLC) in environmental waters.^15^

This scarcity is particularly critical given that the vast majority of existing approaches, even those targeting macrolides, rely on advanced mass spectrometric detection to achieve the required sensitivity.^16^ Such methodologies, although highly sensitive and selective, inherently increase the cost and complexity of analysis, thereby limiting their accessibility and practical application in routine environmental monitoring, especially in laboratories with limited resources.^17^ Furthermore, most of these methods are optimized for specific water matrices, such as surface water, wastewater, or river water, without demonstrating broad applicability across different matrices. A method capable of reliably analyzing varied water types would not only enhance the capacity for water quality monitoring in both urban and rural settings but also facilitate more comprehensive surveillance of environmental antibiotic contamination.^15,17^

Various instrumental techniques have been reported for the quantification of macrolides, including high-performance liquid chromatography coupled with mass spectrometry (LC-MS/MS),^18,19^ HPLC with UV^20^ or PDA^21^ detection, spectrophotometric,^22^ and electrochemical approaches.^23,24^ LC-MS/MS provides excellent sensitivity (typically ng L^−1^ detection) and selectivity but requires expensive instrumentation, highly trained personnel, and extensive sample pretreatment, which limit its routine application in developing regions.^25,26^ Conventional HPLC-UV/PDA methods are simpler and more cost-effective but often exhibit long analysis times, insufficient resolution between co-eluting macrolides, or incomplete validation for complex matrices such as wastewater.^27^ Spectrophotometric and electrochemical methods, although rapid and inexpensive, generally suffer from low selectivity and matrix interference, making them unsuitable for simultaneous multi-analyte quantification.^28,29^

Therefore, it is necessary to find suitable techniques that balance sensitivity, selectivity, simplicity, and affordability. In this context, developing a method that allows reliable detection of multiple macrolides using readily available instrumentation is of significant practical importance.

The present study addresses this gap by developing and validating a simple and economical HPLC-PDA method for the simultaneous determination of azithromycin, clarithromycin, and erythromycin in hospital and livestock wastewater. The method employs an environmentally benign mobile phase composed of acetonitrile and 10 mM NaH_2_PO_4_ buffer (pH 6.0) without the use of toxic modifiers, achieves baseline separation within 7 minutes, and demonstrates satisfactory linearity (2–15 µg mL^−1^), accuracy, and precision. Unlike most reported methods, this approach enables direct analysis of complex wastewater matrices without solid-phase extraction or derivatization, offering a practical alternative for routine environmental monitoring and antibiotic pollution control programs.

Overall, this work provides a balanced analytical strategy combining selectivity and cost-effectiveness, bridging the gap between high-end LC-MS/MS techniques and routine HPLC applications in resource-limited laboratories.

Accordingly, the aim of this study was to develop and validate an optimized HPLC method for the simultaneous, accurate, and sensitive determination of azithromycin, clarithromycin, and erythromycin in environmental water, and to apply it to real samples from local water sources such as untreated hospital influent, near-source discharges from pharmaceutical manufacturing, and highly contaminated livestock ponds to assess contamination levels. Importantly, the proposed method is suitable for laboratories in developing countries such as Vietnam, where advanced instruments like LC-MS/MS or UHPLC-MS/MS are rarely available. To do so, we (i) developed and validated a high-performance liquid chromatography method capable of accurately and sensitively determining azithromycin, clarithromycin, and erythromycin simultaneously in environmental water; (ii) optimized chromatographic parameters to achieve effective resolution, reduced analysis time, and high quantification reliability; and (iii) applied the optimized method to real-world samples to assess contamination levels across multiple local water sources.

## 2. Materials and methods

### 2.1. Chemicals and reagents

All chemicals used in this study were of analytical reagent grade.

Reference standards: ofloxacin (99.95%), norfloxacin (99.85%), and ciprofloxacin (99.95%) were obtained from Merck (Germany). Other reagents including potassium dihydrogen phosphate (KH_2_PO_4_), sodium hydroxide (NaOH), glacial acetic acid (CH_3_COOH, 99.8%), phosphoric acid (H_3_PO_4_, 85%, density = 1.685 g mL^−1^), methanol (MeOH, 100%), acetonitrile (ACN, 99.8%), and triethanolamine (TEA, 100%) were all analytical-grade reagents purchased from Merck (Germany).

### 2.2. Experimental

#### 2.2.1. Instrumentation and detectors

Method development was conducted using an Agilent 1260 Infinity HPLC system equipped interchangeably with UV and photodiode array (PDA) detectors. Both were initially tested; however, the UV detector gave weak and unstable signals, while the PDA detector provided more stable responses and higher selectivity for the three antibiotics (data not provided in the manuscript). Thus, the PDA detector was selected for all analyses.

Preliminary trials were performed using UV detection at several wavelengths (205–215 nm) to assess overall signal response for azithromycin, clarithromycin, and erythromycin. Although the UV detector provided acceptable sensitivity, partial peak overlap and baseline noise were observed in complex wastewater matric.

The PDA detector was subsequently evaluated to improve selectivity and confirm analyte identity based on spectral profiles. PDA detection allowed simultaneous acquisition of absorbance spectra (190–400 nm) for each analyte, facilitating peak purity assessment and reducing matrix interference. Comparative tests demonstrated that the PDA detector provided stronger signal-to-noise ratios and clearer separation among the three macrolides compared with UV detection. Fluorescence and MS detectors were not available in our laboratory.

Based on these evaluations, the PDA detector was selected for all subsequent validation and quantification studies owing to its superior selectivity, reproducibility, and practicality for routine environmental monitoring.

#### 2.2.2. Chromatographic conditions

Chromatographic separation was carried out on an Agilent Zorbax Eclipse C18 column (250 × 4.6 mm, 5 µm) maintained at 35 °C. The mobile phase consisted of acetonitrile and 10 mM NaH_2_PO_4_ buffer (60 : 40, v/v) adjusted to pH 6.0 with dilute phosphoric acid, delivered at a flow rate of 0.8 mL min^−1^. The injection volume was 10 µL. Detection was performed at 210 nm using the PDA detector. Under these optimized conditions, azithromycin, clarithromycin, and erythromycin were completely resolved with symmetrical peaks and baseline separation within 7 minutes.

#### 2.2.3. Optimization of HPLC conditions for the determination of azithromycin, clarithromycin, and erythromycin

In HPLC, the mobile phase typically consists of a mixture of polar and nonpolar solvents, with composition adjusted according to the sample characteristics. Based on literature reports,^30–32^ common solvents for HPLC analysis of macrolide antibiotics include acetonitrile, phosphate buffer, and methanol. In this study, several solvent systems were evaluated to identify the most suitable mobile phase for the simultaneous determination of azithromycin, clarithromycin, and erythromycin by HPLC.

#### 2.2.4. Optimization of HPLC conditions for the determination of azithromycin, clarithromycin, and erythromycin

##### 2.2.4.1 Detector evaluation and selection

Method development was conducted on an Agilent 1260 Infinity HPLC system equipped with interchangeable UV and photodiode array (PDA) detectors. Preliminary experiments using a UV detector at 205–215 nm provided acceptable sensitivity but exhibited partial peak overlap and higher baseline noise in complex wastewater samples. The PDA detector was subsequently tested to improve selectivity and enable simultaneous acquisition of full absorbance spectra (190–400 nm) for each analyte. Comparative tests demonstrated that PDA detection yielded 20–30% higher signal-to-noise ratios and allowed peak purity assessment. Fluorescence and MS detectors were not evaluated, as the focus of this work was to establish a robust, low-cost, and accessible HPLC method for laboratories lacking advanced instrumentation. Based on these results, the PDA detector was selected for all subsequent analyses due to its superior selectivity, reproducibility, and confirmatory spectral capability.

##### 2.2.4.2 Mobile phase optimization

Various binary solvent systems, including acetonitrile/methanol, acetonitrile/water, and acetonitrile/phosphate buffer, were investigated to identify the optimal mobile phase for the simultaneous separation of the target macrolides. The selected system consisted of acetonitrile and 10 mM NaH_2_PO_4_ buffer (60 : 40, v/v), with the buffer adjusted to pH 6.0 using dilute phosphoric acid. This composition provided sharp, symmetrical peaks with good resolution (*R*_s_ > 1.5), stable baselines, and reproducible retention times for all three analytes. Increasing the proportion of acetonitrile (>70%) led to reduced retention and partial co-elution, whereas lower organic content (<50%) resulted in broader peaks and longer analysis times. The optimized mobile phase thus offered the best balance between selectivity, efficiency, and run time.

##### 2.2.4.3 Detection wavelength and retention time

Retention times and detection wavelengths were determined using mixed standard solutions of erythromycin, clarithromycin and azithromycin analyzed under the final optimized chromatographic conditions (Agilent C18 250 × 4.6 mm, 5 µm; 35 °C; acetonitrile: 10 mM NaH_2_PO_4_ pH 6.0 = 60 : 40 v/v; flow 0.8 mL min^−1^; injection volume 10 µL). Retention times (mean of three injections) were 2.724 min (erythromycin), 3.309 min (clarithromycin), and 4.064 min (azithromycin).

A photodiode-array (PDA) detector recorded full UV-Vis spectra across 200–400 nm for each eluted peak. Detection wavelengths for quantification were selected as the absorption maxima observed in the PDA spectra of the pure standards: erythromycin 282.4 nm, clarithromycin 284.0 nm, and azithromycin 288.7 nm. Wavelength selection was performed by overlaying spectra obtained at the front, apex and tail of each chromatographic peak (peak-purity spectra) to confirm spectral consistency and absence of co-eluting interferences. Quantification used the analyte-specific maximum wavelength while peak identity was confirmed by matching both retention time and the PDA spectral fingerprint against reference standards. Flow rate and pH optimization.

The effect of flow rate was evaluated between 0.2–1.0 mL min^−1^, with 0.8 mL min^−1^ chosen as optimal for achieving baseline separation within 7 minutes. The influence of mobile phase pH was studied from 3.0 to 9.0; pH 6.0 produced the most stable retention times and best peak resolution.

##### 2.2.4.4 Optimized chromatographic conditions

Chromatographic separation was performed on an Agilent Zorbax Eclipse C18 column (250 × 4.6 mm) maintained at 35 °C. The mobile phase consisted of acetonitrile and 10 mM NaH_2_PO_4_ buffer (60 : 40, v/v, pH 6.0), delivered at a flow rate of 0.8 mL min^−1^. The injection volume was 10 µL. Detection was performed at 210 nm using the PDA detector. Under these conditions, the three analytes were completely resolved with *R*_s_ > 1.5.

#### 2.2.5. Method validation

##### 2.2.5.1 System suitability testing

Before each analytical run, system suitability was verified to ensure the performance and stability of the HPLC system. A mixed standard solution containing azithromycin, clarithromycin, and erythromycin at mid-range concentrations was injected six times, and the following parameters were evaluated in accordance with USP/ICH guidelines:

• Retention time (*t*_r_): used to confirm the reproducibility of analyte elution. The RSD of retention time values should be ≤ 1%.

• Resolution (*R*_s_): indicates separation between adjacent peaks, calculated as:*R*_s_ = 2(*t*_R2_ − *t*_R1_)/*W*_1_ + *W*_2_where *t*_R1_ and *t*_R2_ are the retention times of two consecutive peaks, and *W*_1_ and *W*_2_ are their baseline widths. An *R*_s_ value ≥1.5 was considered acceptable.

• Column efficiency (*N*): calculated from the azithromycin peak as:*N* = 16(*t*_R_/*W*)^2^where *W* is the baseline peak width. Theoretical plate counts greater than 2000 indicate adequate column performance.

• Tailing factor (*T*_f_): measures peak symmetry, calculated as:*T*_f_ = *W*_0.05_/2*f*where *W*_0.05_ is the peak width at 5% of height and *f* is the distance from the peak maximum to the front of the peak. Acceptable values range from 0.9 to 1.5.

The %RSD of retention time and peak area for all analytes was below 2%, and *R*_s_ values between adjacent peaks exceeded 1.5, confirming adequate resolution. The theoretical plate number (*N*) values exceeded 4000, and tailing factors ranged from 1.02 to 1.18, indicating excellent column efficiency and peak symmetry. These results confirm that the HPLC system was suitable for routine analysis.

A standard solution of the three macrolides was analyzed six consecutive times to evaluate repeatability. Retention time and peak area were used to calculate relative standard deviation (RSD) as indicators of system precision.

As summarized in Table 1, all parameters met USP/ICH acceptance criteria, with *R*_s_ values greater than 2.0 for all peak pairs, *t*_R_ %RSD less than 1%, plate numbers exceeding 2000, tailing factors between 0.8 and 1.5, and peak area %RSD values below 2%. These results confirm that the HPLC system was suitably stable, reproducible, and fit for routine quantitative analysis.

**Table 1:** System suitability parameters (*t*_R_, *R*_s_, *N*, *T*_f_, %RSD) for the three macrolides based on six replicate standard injections under optimized HPLC-PDA conditions^a^

Parameter	Erythromycin	Clarithromycin	Azithromycin	Acceptance criteria (USP/ICH)
Retention time (*t*_R_, min)	2.771 ± 0.027	3.344 ± 0.028	4.119 ± 0.001	
%RSD (*t*_R_)	0.968	0.842	0.034	%RSD < 1%
Peak area %RSD (*n* = 6)	0.216	0.188	0.269	<2%
Resolution (*R*_s_)	—	2.09 ± 0.02	2.23 ± 0.02	>2.0
Tailing factor (*T*_f_)	1.06 ± 0.02	1.07 ± 0.01	1.10 ± 0.01	0.8–1.5
Plate number (*N*)	2120 ± 24	2583 ± 67	2808 ± 64	>2000

a System suitability results obtained from six replicate injections of the mixed macrolide standard solution under optimized chromatographic conditions. Parameters include retention time (*t*_R_), peak area repeatability (%RSD), resolution between adjacent peaks (*R*_s_), theoretical plate number (*N*), and tailing factor (*T*_f_). All values comply with USP/ICH acceptance criteria, confirming adequate performance and stability of the HPLC system prior to sample analysis.

A standard solution with a known analyte concentration was analyzed nine consecutive times using HPLC. The relative standard deviation (RSD) of retention time and peak area was calculated as follows:

Mean value:1
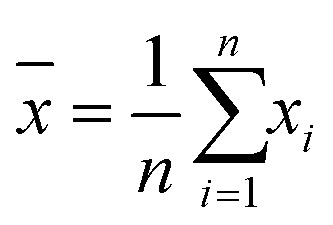
where *x̄* is the arithmetic mean and *x*_*i*_ represents each measurement.

Standard deviation (SD):2
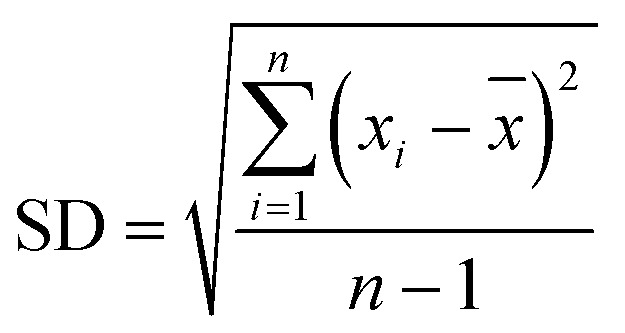


Relative standard deviation (RSD):3
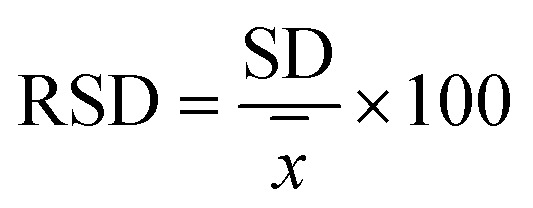


#### 2.2.6. Limit of detection (LOD) and limit of quantification (LOQ)

The LOD was defined as the lowest analyte concentration giving a signal three times higher than the baseline noise, calculated using eqn (4):4
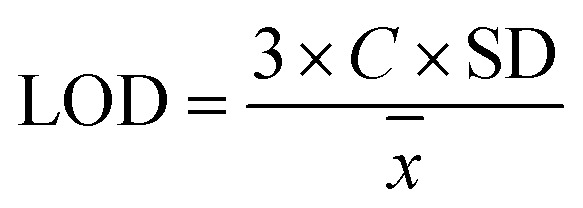
where *C* is the concentration of the standard solution.^33^

The LOQ was defined as the lowest concentration quantified with acceptable precision (S/N ≥ 10), calculated using eqn (5):5
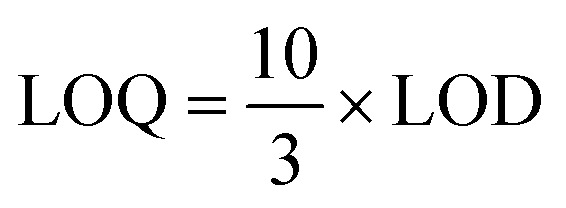
where slope is expressed as peak area per unit concentration (*e.g.*, area/ppm).^33^

#### 2.2.7. Accuracy (recovery)

Accuracy was evaluated using the standard addition method at three concentration levels within the validated range for each analyte. Known amounts (3, 2 and 2 µg mL^−1^) of azithromycin, clarithromycin, and erythromycin, respectively, were spiked into wastewater samples to achieve final concentrations of approximately 6, 6, and 10 µg mL^−1^, representing medium, and high levels, respectively. Each spiked sample was analyzed in triplicate under the optimized chromatographic conditions.

Recovery (*R*%) was calculated using eqn (6):

6 *R*% = (*C*_m+c_ − *C*_m_)/*C*_c_ × 100

where *C*_m+c_ is the analyte concentration in the spiked sample, *C*_m_ is the concentration in the unspiked sample, and *C*_c_ is the added concentration.

The mean recoveries for azithromycin, clarithromycin, and erythromycin ranged from 99.1% to 104.5%, with %RSD values almost below 2%, demonstrating excellent accuracy and precision of the developed HPLC-PDA method.

### 2.3. Sample collection and preparation

#### 2.3.1. Sampling procedure

In this study, wastewater samples were collected from hospitals and livestock farms at selected sites in Cao Bang and Ha Nam provinces, in Vietnam. Sampling was conducted in the field at a depth of approximately 10 cm below the water surface. For each site, around 500 mL of wastewater was collected into pre-cleaned plastic bottles that had been rinsed with the target sample prior to collection. Samples were placed in insulated containers with ice for transport, and all relevant information was recorded on-site. Upon arrival at the laboratory, samples were stored under refrigerated conditions at 4 °C and for 24 h until analysis. The location of the sample was described in Table S1 (SI).

#### 2.3.2. Sample preparation for analysis

After allowing the samples to settle, the supernatant was decanted and centrifuged at 4000 rpm for 5 minutes using a laboratory centrifuge. The resulting supernatant was then filtered through a 0.22 µm Whatman membrane filter. The filtrate was analyzed by high-performance liquid chromatography (HPLC) equipped with a photodiode array (PDA) detector, using a flow rate of 0.8 mL min^−1^. Identification and quantification of the analytes were based on their retention times and peak areas.

### 2.4. Statistics

All experimental results are expressed as mean values accompanied by their standard deviations (mean ± SD). Depending on the design, each treatment was carried out either in triplicate or in six independent replicates. Statistical analyses and graphical representations were performed using Origin pro 2019. Comparisons among groups were assessed through one-way analysis of variance (ANOVA) or independent-sample *t*-tests, as appropriate. When significant differences were detected, multiple comparisons of group means were conducted using Tukey's Honestly Significant Difference (HSD) test. A threshold of *p* < 0.05 was set to indicate statistical significance.

## 3. Results and discussion

### 3.1. Optimization of HPLC parameters

#### 3.1.1. Selection of detector

**Fig. 1:**
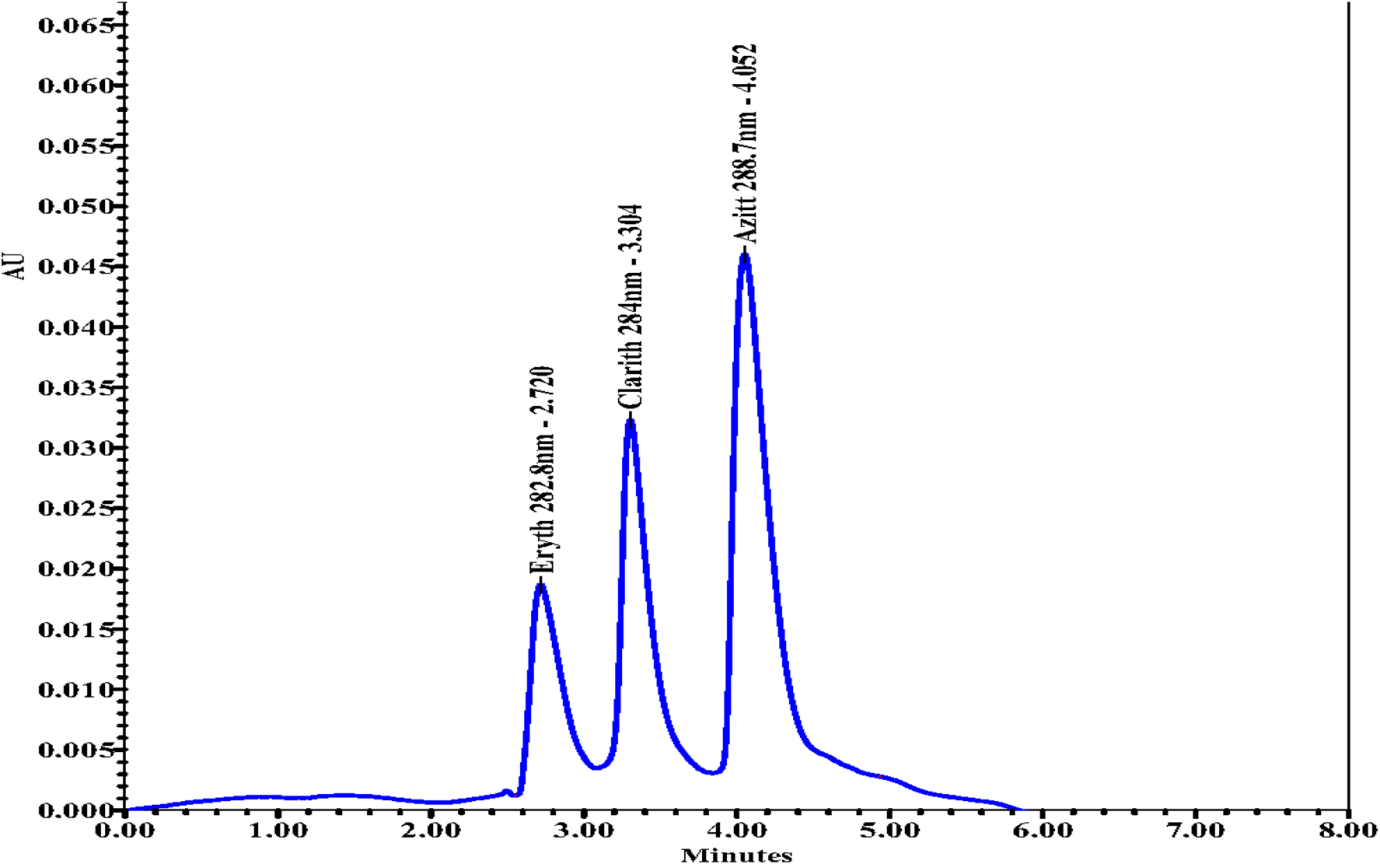
Chromatographic separation of azithromycin, clarithromycin, and erythromycin using a PDA detector. Chromatographic conditions: Agilent Zorbax Eclipse C18 column (250 × 4.6 mm, 5 µm) at 35 °C; mobile phase acetonitrile/10 mM NaH_2_PO_4_ buffer (60 : 40, v/v, pH 6.0); flow rate 0.8 mL min^−1^; injection volume 10 µL; detection at 210 nm using PDA detector.

Since the Photodiode Array (PDA) detector offers several advantages over the conventional UV-Vis detector, the PDA was selected for chromatographic analysis of the standard mixture containing azithromycin, clarithromycin, and erythromycin. The resulting chromatogram is presented in Fig. 1.

The results demonstrate that the analytes were completely resolved, confirming the suitability of PDA detection for this analysis. In method development for HPLC, an essential initial step was the evaluation of appropriate detectors. Both UV-Vis and PDA detectors were tested. Compared to UV-Vis, the PDA provided more stable signals, a cleaner baseline, and sharper, well-defined peaks.

A key advantage of PDA detection lies in its ability to simultaneously capture multi-wavelength absorption spectra (200–400 nm), enabling compound identification based on characteristic spectra while minimizing peak misassignment. These findings are consistent with previous reports,^34,35^ where PDA was shown to outperform UV-Vis for antibiotic analysis. Therefore, PDA was chosen for all subsequent experiments.

#### 3.1.2. Selection of mobile phase

To optimize the mobile phase, several solvent systems were evaluated, including acetonitrile, phosphate buffer, methanol, and water. Several volumetric ratios of acetonitrile (organic phase) and 10 mM NaH_2_PO_4_ buffer (aqueous phase, pH 6.0) were tested to determine the optimal mobile phase composition for simultaneous separation of azithromycin, clarithromycin, and erythromycin. Ratios of 40 : 60, 50 : 50, 60 : 40, 65 : 35, and 70 : 30 (v/v) were systematically evaluated. Increasing the proportion of acetonitrile shortened the retention times but slightly reduced peak resolution, while lower acetonitrile content resulted in broader peaks and longer analysis times. The ratio of 60 : 40 (v/v) acetonitrile to 10 mM NaH_2_PO_4_ buffer provided the best compromise between analysis time, resolution (*R*_s_ > 1.5), and peak symmetry. This composition was therefore selected as the final mobile phase for all subsequent analyses.

**Fig. 2:**
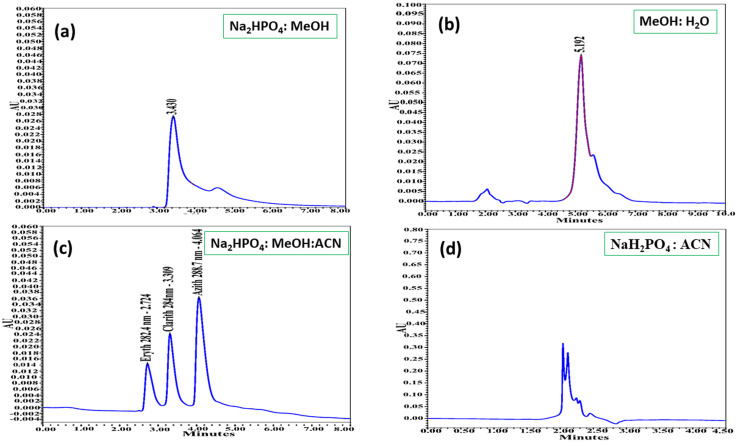
Chromatographic separation of azithromycin, clarithromycin, and erythromycin using different mobile phases: (a) NaH_2_PO_4_/MeOH, (b) NaH_2_PO_4_/MeOH/ACN, (c) MeOH/H_2_O, and (d) NaH_2_PO_4_/ACN. Representative chromatograms of standard solutions of azithromycin (1), clarithromycin (2), and erythromycin (3) obtained under the optimized HPLC-PDA conditions. Chromatographic conditions: Agilent Zorbax Eclipse C18 column (250 × 4.6 mm, 5 µm) maintained at 35 °C; mobile phase of acetonitrile and 10 mM NaH_2_PO_4_ buffer (60 : 40, v/v, pH 6.0); flow rate 0.8 mL min^−1^; injection volume 10 µL; detection at 210 nm using PDA detector. Retention times: 3.76 min (azithromycin), 4.91 min (clarithromycin), and 6.23 min (erythromycin).

Chromatographic analyses were performed using a mixed standard solution of azithromycin (15 mg L^−1^), clarithromycin (10 mg L^−1^), and erythromycin (7 mg L^−1^). The tested mobile phase compositions were: NaH_2_PO_4_/MeOH, NaH_2_PO_4_/MeOH/ACN, MeOH/H_2_O, and NaH_2_PO_4_/ACN. The corresponding chromatograms are presented in Fig. 2.

The results indicated that the acetonitrile/phosphate buffer system provided the most suitable mobile phase for this analysis. Under these conditions, the peaks of erythromycin, clarithromycin, and azithromycin were sharp, well-resolved, and symmetrical, in contrast to the broader or less stable peaks obtained with methanol-based systems.

These findings are consistent with previous studies highlighting the effectiveness of acetonitrile in combination with phosphate buffer for macrolide analysis, due to its low viscosity, high resolution, and stable pressure on C18 columns.^36,37^ The reduced viscosity of the ACN/phosphate buffer mixture facilitates smooth flow and minimizes backpressure, thereby improving column stability and ensuring reliable performance in extended chromatographic runs.^38,39^

#### 3.1.3. Evaluation of retention time and absorption wavelengths

The retention behavior and characteristic absorption wavelengths of the target analytes were investigated using the optimized detector and mobile phase conditions. A mixed standard solution of azithromycin (15 mg L^−1^), clarithromycin (10 mg L^−1^), and erythromycin (7 mg L^−1^) was analyzed over a 30 minutes run within the wavelength range of 200–400 nm. The results are presented in Fig. 1.

Retention times were established using mixed standard solutions under the final optimized chromatographic conditions and are 2.724 min (erythromycin), 3.309 min (clarithromycin) and 4.064 min (azithromycin).

Detection wavelengths for quantification were selected from PDA full spectral scans (200–400 nm) of the pure standards and correspond to the absorption maxima of each compound: erythromycin 282.4 nm, clarithromycin 284.0 nm, and azithromycin 288.7 nm.

Wavelength selection was confirmed by overlaying spectra from different portions of each chromatographic peak (leading edge, apex, tail) to verify spectral purity and absence of co-eluting components. Quantification was performed at the analyte-specific maxima while identification was confirmed by matching both retention time and PDA spectral fingerprint.

#### 3.1.4. Investigation of mobile phase ratios

**Fig. 3:**
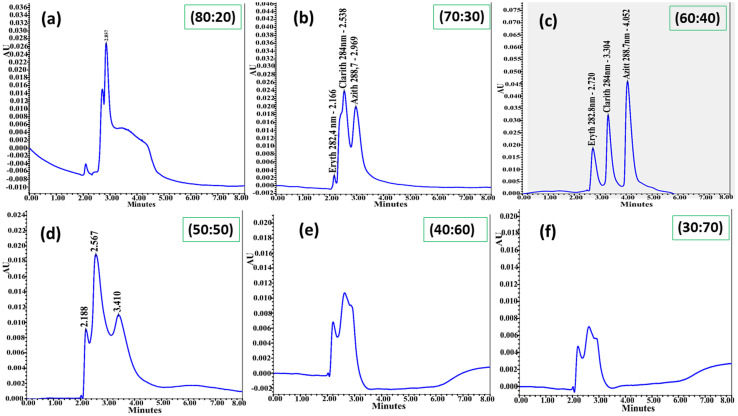
Chromatograms of azithromycin, clarithromycin, and erythromycin obtained using different volume ratios of acetonitrile and phosphate buffer as the mobile phase: (a) 80 : 20, (b) 70 : 30, (c) 60 : 40, (d) 50 : 50, (e) 40 : 60, (f) 30 : 70 (v/v). Chromatographic conditions: Agilent Zorbax Eclipse C18 column (250 × 4.6 mm, 5 µm) maintained at 35 °C; flow rate 0.8 mL min^−1^; injection volume 10 µL; detection using PDA detector.

After identifying acetonitrile and phosphate buffer as the most suitable mobile phase components, different volume ratios of ACN : NaH_2_PO_4_ were tested (90 : 10, 80 : 20, 70 : 30, 60 : 40, 50 : 50, 40 : 60, 30 : 70, 20 : 80, and 10 : 90, v/v) to determine the optimal composition for analysis. The results of these experiments are presented in Fig. 3 and summarized in Table 2.

**Table 2:** Effect of the ACN : NaH_2_PO_4_ ratio on the retention time and peak area of azithromycin, clarithromycin, and erythromycin^a^

Ratio of ACN : NaH_2_PO_4_	ERYTH		CLARITH		AZITH	
	Retention time (min)	Peak area (m Au)	Retention time (min)	Peak area (m Au)	Retention time (min)	Peak area (m Au)
90/10	2.175	2039	ND	ND	ND	ND
80/20	ND	ND	ND	ND	2.858	7203
70/30	2.166	1092	2.538	8039	2.969	6029
60/40	2.724	6720	3.309	8018	4.064	18 203
50/50	2.185	1782	2.568	6217	3.410	8038
40/60	ND	ND	ND	ND	ND	ND
30/70	ND	ND	ND	ND	ND	ND
20/80	ND	ND	ND	ND	ND	ND
10/90	ND	ND	ND	ND	ND	ND

a ND: no detection.

The results showed that most ACN : NaH_2_PO_4_ ratios (90 : 10, 80 : 20, 70 : 30, 40 : 60, 30 : 70, 20 : 80, and 10 : 90, v/v) produced either poorly resolved peaks or no detectable peaks at all. At ratios of 50 : 50 and 60 : 40 (v/v), the analytes were separated to varying degrees. With the 50 : 50 ratio, the peaks appeared close together with short retention times, suggesting incomplete resolution. In contrast, the 60 : 40 ratio yielded well-resolved, symmetrical, and balanced peaks with complete separation of the three analytes. Therefore, the mobile phase composition of ACN : NaH_2_PO_4_ (60 : 40, v/v) was selected as the optimal condition for subsequent analyses.

#### 3.1.5. Investigation of flow rate

**Fig. 4:**
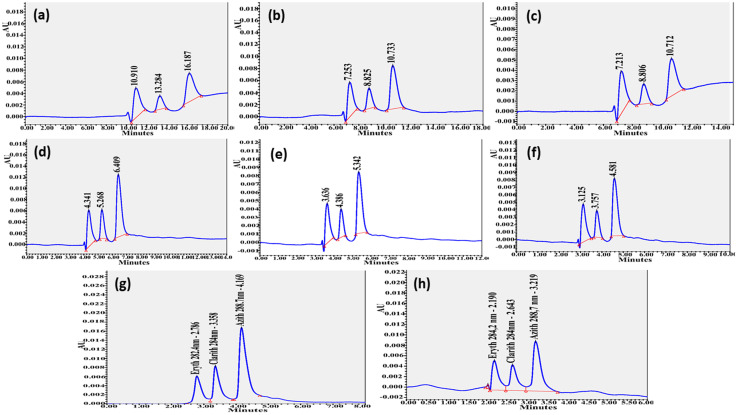
Chromatograms of azithromycin, clarithromycin, and erythromycin obtained at different flow rates: (a) 0.2 mL min^−1^, (b) 0.3 mL min^−1^, (c) 0.4 mL min^−1^, (d) 0.5 mL min^−1^, (e) 0.6 mL min^−1^, (f) 0.7 mL min^−1^, (g) 0.8 mL min^−1^, and (h) 0.9 mL min^−1^. Chromatographic conditions: Agilent Zorbax Eclipse C18 column (250 × 4.6 mm, 5 µm) maintained at 35 °C; mobile phase of acetonitrile and 10 mM NaH_2_PO_4_ buffer (60 : 40, v/v, pH 6.0); injection volume 10 µL; using PDA detector.

The effect of flow rate was examined in the range of 0.2–1.0 mL min^−1^ under the optimized detector and mobile phase conditions. The chromatograms obtained at different flow rates are shown in Fig. 4, and the results are summarized in Table 3.

**Table 3:** Effect of flow rate on the retention time and peak area of erythromycin, clarithromycin, and azithromycin

Flow rate (mL min^−1^)	ERYTH		CLARITH		AZITH	
	Retention time (min)	Peak area (m Au)	Retention time (min)	Peak area (m Au)	Retention time (min)	Peak area (m Au)
0.2	10.898	6554	13.292	6564	16.185	16 254
0.3	7.264	6598	8.824	6124	10.744	16 465
0.4	7.231	6645	8.798	6624	10.712	17 123
0.5	4.337	6578	5.265	7869	6.409	17 109
0.6	3.632	6684	4.393	7901	5.347	17 689
0.7	3.126	6432	3.764	7954	4.585	17 032
0.8	2.724	6720	3.309	8018	4.064	18 203
0.9	2.403	6514	2.929	8024	3.563	17 654
1.0	2.190	6741	2.643	7584	3.219	1624

The results indicated that at lower flow rates (0.2–0.4 mL min^−1^), the analytes exhibited relatively long retention times, while peak areas showed a slight but not significant decrease. The peaks remained sharp and symmetrical. At moderate flow rates (0.5–0.8 mL min^−1^), retention times were reduced, and peak areas decreased more rapidly, although peak symmetry was still maintained. At higher flow rates (0.9–1.0 mL min^−1^), retention times shortened further, but peak bases became broader and less stable. These observations can be explained by the chromatographic behavior of analytes at different flow rates. At low flow rates, analytes migrate more slowly through the column, resulting in longer contact with the stationary phase, stronger interactions, and consequently larger retention times and peak areas. As the flow rate increases, analytes pass through the column more quickly, reducing interaction with the stationary phase, which shortens retention time and decreases peak area.

To balance analysis time, resolution, and peak symmetry, a flow rate of 0.8 mL min^−1^ was selected as the optimal condition for subsequent measurements.

#### 3.1.6. Investigation of mobile phase pH

**Fig. 5:**
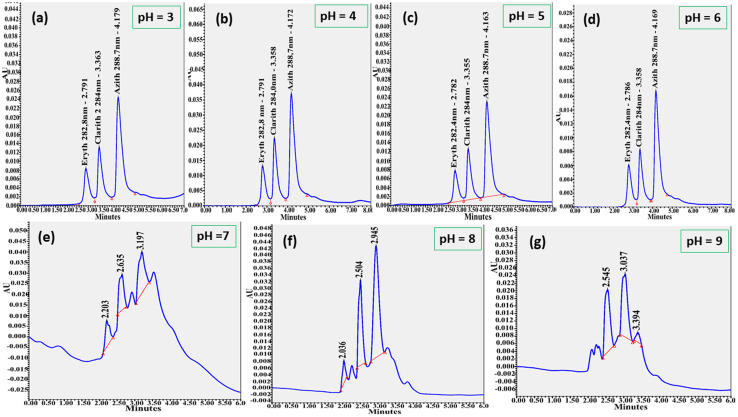
Chromatograms of azithromycin, clarithromycin, and erythromycin at pH 3 (a), pH 4 (b), pH 5 (c), pH 6 (d), pH 7 (e), pH 8 (f), and pH 9 (g).

The influence of mobile phase pH was investigated at values of 3.0, 4.0, 5.0, 6.0, 7.0, 8.0, and 9.0. Chromatograms were recorded for a mixed standard solution containing azithromycin (15 mg L^−1^), clarithromycin (10 mg L^−1^), and erythromycin (7 mg L^−1^) under different pH conditions, with a flow rate of 0.8 mL min^−1^ and an injection volume of 10.0 µL. The results are shown in Fig. 5 and summarized in Table 4.

**Table 4:** Effect of mobile phase pH on the retention time and peak area of erythromycin, clarithromycin, and azithromycin

pH	Retention time (min)			Peak area (m Au)		
	ERYTH	CLARITH	AZITH	ERYTH	CLARITH	AZITH
3.0	2.791	3.360	4.176	6903	9908	15 123
4.0	2.791	3.358	4.172	7401	9801	16 720
5.0	2.782	3.355	4.163	7914	11 100	18 023
6.0	2.186	3.351	4.166	8159	11 923	18 013
7.0	2.270	2.663	3.237	7986	9645	16 098
8.0	2.225	2.595	3.143	6212	9912	13 903
9.0	2.549	3.037	3.395	6012	6354	2134

The results indicated that as the mobile phase pH increased from 3.0 to 6.0, the retention times of the analytes remained nearly unchanged, while peak areas gradually increased. The peaks were sharp, symmetrical, and stable under these conditions. However, when the pH was further increased from 7.0 to 9.0, retention times decreased, peak bases became broader, and asymmetry was observed. This can be attributed to the ionization of analyte molecules at higher pH, which reduces their interaction with the stationary phase, leading to shorter retention and smaller peak areas. Moreover, in alkaline environments (pH > 7), the silica backbone of the stationary phase may undergo partial dissolution (SiO_2_ forming silicates), resulting in reduced peak areas, loss of bonded phase ligands, peak broadening, and diminished chromatographic performance. Furthermore, the stabilization and resolution of macrolide molecules can indeed be improved with the inclusion of a phosphate buffer at pH 6.0. This pH minimizes secondary ionic interactions from silica surface silanol groups, and buffered pH conditions have been shown to enhance chromatographic performance.^40^ For these reasons, and to ensure both accurate analysis of erythromycin, clarithromycin, and azithromycin and protection of the chromatographic column, a mobile phase pH of 6.0 was selected as the optimal condition.

Based on the systematic evaluation of detector type, mobile phase composition and ratio, flow rate, and pH, the optimal chromatographic conditions for the simultaneous determination of erythromycin, clarithromycin, and azithromycin were established. The photodiode array (PDA) detector provided superior sensitivity and selectivity compared to UV-Vis, while acetonitrile combined with phosphate buffer ensured efficient separation with stable baselines and symmetrical peaks. A mobile phase ratio of ACN : NaH_2_PO_4_ (60 : 40, v/v) at a flow rate of 0.8 mL min^−1^ was found to deliver the best balance between resolution, retention, and analysis time. Furthermore, maintaining the buffer at pH 6.0 prevented peak distortion and safeguarded column stability. The optimized experimental parameters are summarized in Table 5.

**Table 5:** Optimized experimental conditions for the simultaneous determination of erythromycin, clarithromycin, and azithromycin by HPLC

Parameter	Optimized value
Detector type	PDA
Column (stationary phase)	Agilent C18 (250 mm × 4.6 mm, 5 µm)
Mobile phase	ACN : NaH_2_PO_4_ (v/v)
Mobile phase ratio (v/v)	ACN : NaH_2_PO_4_ = 60 : 40 (v/v)
Flow rate	0.8 mL min^−1^
Buffer composition/concentration	10 mM NaH_2_PO_4_
Buffer pH	6.0
Column temperature	35 °C
Injection volume	10 µL

### 3.2. Chromatographic conditions

Chromatographic separation was carried out on an Agilent 1260 Infinity HPLC system equipped with a photodiode array detector (PDA). An Agilent Zorbax Eclipse C18 column (250 × 4.6 mm, 5 µm) maintained at 35 °C was used. The mobile phase consisted of acetonitrile and 10 mM NaH_2_PO_4_ buffer (60 : 40, v/v) adjusted to pH 6.0 with dilute phosphoric acid, delivered at a flow rate of 0.8 mL min^−1^. The injection volume was 10 µL, and detection was performed at erythromycin 282.4 nm, clarithromycin 284.0 nm, and azithromycin 288.7 nm, respectively. Under these optimized conditions, azithromycin, clarithromycin, and erythromycin were completely resolved with symmetrical peaks and baseline separation within 7 minutes.

### 3.3. Validation results

#### 3.3.1. System suitability

Under the optimized chromatographic conditions established above, the system suitability was evaluated by repeated injection of a standard mixture containing azithromycin (15 mg L^−1^), clarithromycin (10 mg L^−1^), and erythromycin (7 mg L^−1^). Each solution was analyzed nine consecutive times, and the relative standard deviations (RSD%) of the analyte peak areas were calculated to assess method precision and instrument performance. The results are summarized in Table 6.

**Table 6:** Results of system suitability test calculations

Number of injections	Retention time (min)			Peak area (m Au)		
	ERYTH	CLARITH	AZITH	ERYTH	CLARITH	AZITH
1 (*x*_1_)	2.786	3.350	4.166	6723	8012	18 203
2 (*x*_2_)	2.724	3.309	4.064	6724	8013	18 203
3 (*x*_3_)	2.783	3.337	4.166	6724	8013	18 204
4 (*x*_4_)	2.791	3.358	4.172	6725	8013	18 207
5 (*x*_5_)	2.781	3.356	4.170	6727	8012	18 208
6 (*x*_6_)	2.782	3.357	4.170	6728	8016	18 203
7 (*x*_7_)	2.783	3.356	4.170	6723	8012	18 201
8 (*x*_8_)	2.782	3.355	4.170	6723	8013	18 203
9 (*x*_9_)	2.781	3.358	4.170	6723	8012	18 203
*X*_tb_	2.777	3.348	4.158	6724.4	8012.9	18 203.9
SD	0.020	0.016	0.035	1.830	1.269	2.205
RSD (%)	0.720	0.478	0.842	0.027	0.016	0.012

The calculated RSD (%) values of both retention time and peak area for the analytes across nine replicate injections were in the range of 0.012–0.842%, which is well below the generally accepted threshold of 2% (AOAC). This clearly demonstrates that the chromatographic system operated with excellent repeatability and minimal variability. Such low RSD values confirm not only the stability of the system but also its robustness under the optimized experimental conditions. Consequently, the method can be considered highly reliable for routine analysis of erythromycin, clarithromycin, and azithromycin in complex sample matrices.

#### 3.3.2. Linearity range

**Fig. 6:**
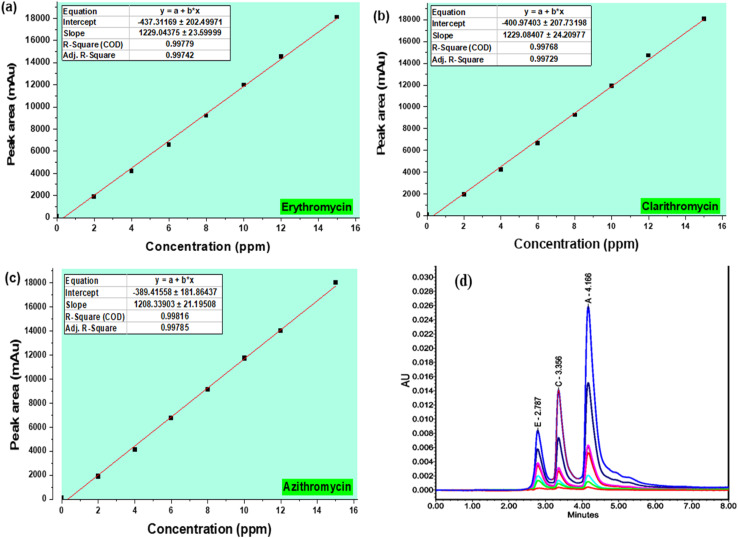
Calibration curves showing the linear relationship between concentration and peak area for erythromycin (a), clarithromycin (b), and azithromycin (c). Overlaid chromatographic profiles at 2, 4, 6, 8, 10, 12, and 15 mg L^−1^ are also presented for all analytes (d). Chromatographic conditions: Agilent Zorbax Eclipse C18 column (250 × 4.6 mm, 5 µm) maintained at 35 °C; mobile phase of acetonitrile and 10 mM NaH_2_PO_4_ buffer (60 : 40, v/v, pH 6.0); flow rate 0.8 mL min^−1^; injection volume 10 µL; detection at 210 nm using PDA detector. Retention times: 3.76 min (azithromycin), 4.91 min (clarithromycin), and 6.23 min (erythromycin).

Calibration curves for erythromycin, clarithromycin, and azithromycin were constructed using standard solutions at seven concentration levels within the range of 2–15 ppm (or mg L^−1^) (Table 7). The regression equations obtained showed good linearity, with correlation coefficients (*R*^2^) exceeding 0.997 for all analytes (Fig. 6, Table 7). These results indicate that the developed HPLC method provides an excellent linear response across the investigated concentration ranges, ensuring its suitability for quantitative determination of the three macrolide antibiotics in complex matrices.

**Table 7:** Results of the calibration curve linearity for ERYTH, CLARITH, and AZITH

ERYTH		CLARITH		AZITH	
Concentration (ppm)	Peak area (mAu)	Concentration (ppm)	Peak area (mAu)	Concentration (ppm)	Peak area (mAu)
2	1892	2	1942	2	1902
4	4210	4	4210	4	4120
6	6581	6	6651	6	6731
8	9188	8	9248	8	9128
10	11 983	10	11 923	10	11 723
12	14 503	12	14 703	12	14 033
15	18 100	15	18 053	15	18 013
*y* = 1229.0*x* − 437.3		*y* = 1229.1*x* − 400.9		*y* = 1208.3*x* − 389.4	
*R* ^2^ = 0.9978		*R* ^2^ = 0.9977		*R* ^2^ = 0.9982	

Representative chromatograms of AZM, CLM, and ERY at increasing concentrations (2.00–15.0 mg L^−1^) are shown in Fig. 6. The chromatograms demonstrate a clear proportional increase in peak area with increasing concentration while retention times remained constant, confirming the linear response and sensitivity of the developed method. The corresponding calibration curves (inset in Fig. 6) exhibited excellent linearity, with correlation coefficients (*R*^2^) above 0.997 for all analytes. These results validate the suitability of the HPLC-PDA method for simultaneous quantification of macrolide antibiotics in environmental matrices.

From the calibration curve data, the regression coefficients (*R*^2^) for erythromycin, clarithromycin, and azithromycin were all within the acceptable range of 0.99–1.00. This clearly demonstrates an excellent linear correlation between peak area and analyte concentration across the investigated ranges, confirming that the developed method provides reliable quantitative performance for simultaneous determination of the three macrolides.

#### 3.3.3. Limit of detection (LOD) and limit of quantification (LOQ) of the instrument

To determine the detection limit, nine replicate injections of the mixed standard solution containing azithromycin (15 ppm), clarithromycin (10 ppm), and erythromycin (7 ppm) were performed. Based on the measurement results, the LOD values of the analytes were calculated according to eqn (4) and are presented in Table 8. Subsequently, the LOQ values were calculated from the LOD results using equation (eqn (5)), as shown in Table 7. Accordingly, the lowest quantifiable concentrations of erythromycin, clarithromycin, and azithromycin by the HPLC system were 0.020 ppm, 0.017 ppm, and 0.017 ppm (or 20, 17 and 17 µg L^−1^), respectively.

**Table 8:** Limits of detection (LOD) and quantification (LOQ) of the method

Substance	Concentration *C* (ppm)	Standard deviation (SD)	Mean value (*X̄*)	Limit of detection LOD (µg L^−1^)	Limit of quantity LOQ (µg L^−1^)
ERYTH	7	1.830	6724.4	6.0	20.0
CLARITH	10	1.269	8012.9	5.0	17.0
AZITH	15	2.205	18 203.9	5.0	17.0

The LODs of 5–6 µg L^−1^ for azithromycin, clarithromycin, and erythromycin are higher than typical concentrations in environmental waters, which are often in the ng L^−1^ range. These values were higher than those of other methods such as UHPLC-MS/MS (LOD = 0.4 µg L^−1^)^41^ and LC-MS/MC (LOD = 0.04–0.90 ng L^−1^).^42^ However, this HPLC-PDA method was developed for routine monitoring of wastewater samples, where macrolide concentrations are generally higher (up to several µg L^−1^, as observed in our hospital and livestock wastewater samples). Many laboratories in our country have access only to conventional HPLC instruments and not to advanced systems like HPLC-MS/MS or LC-MS/MS, which can achieve ng L^−1^ detection limits. Therefore, despite not reaching ng L^−1^ sensitivity, the method is practical, cost-effective, and well-suited for the conditions of many laboratories in our country, providing reliable data for screening and monitoring higher-concentration samples.

Although the developed HPLC-PDA method demonstrated excellent precision, linearity (*R*^2^ ≥ 0.997), and accuracy (recoveries 99.75–104.53%), its instrumental detection limits (LOD = 5–6 µg L^−1^) are higher than the ng L^−1^ levels typically reported for surface waters. This limitation arises from the intrinsic sensitivity of UV-based detection compared with LC-MS/MS systems. Nevertheless, the current method was designed for the routine quantification of macrolides in untreated hospital and livestock wastewater, where the analyte concentrations are in the µg L^−1^ to mg L^−1^ range,^43,44^ well above the LOD. The absence of detectable interferences and the strong reproducibility confirm its suitability for this purpose.

At present, our laboratory does not possess SPE facilities or LC-MS/MS instrumentation; thus, additional pre-concentration steps and ultra-trace validation could not be performed. This limitation is explicitly acknowledged, and future work will aim to collaborate with well-equipped laboratories to extend the method's applicability toward ng L^−1^ detection. Despite these constraints, the proposed HPLC-PDA method remains a robust, cost-effective, and accessible approach for the continuous monitoring of macrolide antibiotics in wastewater treatment systems and pollution-source tracking.

#### 3.3.4. Recovery

**Fig. 7:**
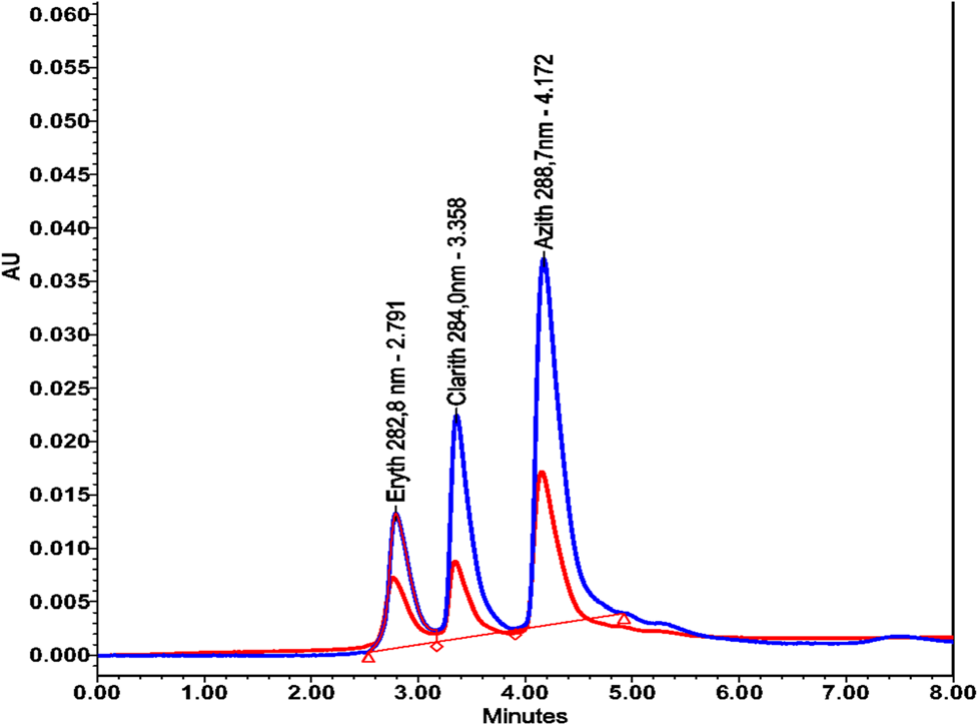
Chromatograms of erythromycin (ERYTH), clarithromycin (CLARITH), and azithromycin (AZITH) before (red line) and after spiking into sample S11 (blue line).

To evaluate the recovery, the real sample S11 was first diluted by adding 7 mL of double-distilled water into a 100 mL volumetric flask and then filling up to the mark with the S11 solution. For the spiked sample, 2 mL of erythromycin (100 ppm), 2 mL of clarithromycin (100 ppm), and 3 mL of azithromycin (100 ppm) were added to a 100 mL volumetric flask, followed by dilution to the mark with the S11 solution. The recovery was assessed at one concentration level (medium) within the validated range, which were 2, 2 and 3 µg mL^−1^ spiked in the real sample. Both the diluted and spiked S11 samples were analyzed by HPLC. Based on the chromatographic results, calibration curves, and eqn (6), the recovery rates were calculated. The results are presented in Table 9 and Fig. 7.

**Table 9:** Recovery test results for ERYTH, CLARITH, and AZITH in real sample S11 (standard addition method)

Analyte	Peak area in real samples (m Au)	Peak area in spiked real sample (m Au)	Concentration in real sample *C*_m_ (ppm)	Concentration in spiked real SampleC_m+c_ (ppm)	Recovery (R%)
ERYTH	4510	6964	4.045	6.040	99.75
CLARITH	4344	6845	3.885	5.912	101.35
AZITH	8290	12 094	7.194	10.33	104.53

The recovery results demonstrate that the developed HPLC method provides accurate quantification of erythromycin, clarithromycin, and azithromycin in real water samples. The calculated recovery values fall within the generally accepted range (typically 80–120%), indicating that matrix effects do not significantly interfere with the determination. Furthermore, the close agreement between the measured and theoretical concentrations confirms the robustness of the calibration model. Overall, these findings highlight that the proposed method is both reliable and suitable for routine monitoring of macrolide antibiotics in environmental samples.

#### 3.3.5. Precision (intra-day and inter-day repeatability)

The precision of the developed HPLC-PDA method was evaluated in accordance with ICH Q2 (R2) and AOAC guidelines by assessing both intra-day (repeatability) and inter-day (intermediate precision) variations.

For intra-day precision, mixed standard solutions containing azithromycin, clarithromycin, and erythromycin at the concentration level of 5, 7, and 10 µg mL^−1^, respectively, were analyzed in five times within the same day under identical chromatographic conditions. For inter-day precision, the same procedure was repeated over five consecutive days, using freshly prepared standards each day. The %RSD of retention times and peak areas was calculated for each analyte at each concentration level.

The obtained %RSD values for both intra- and inter-day measurements were below 2% for all analytes, indicating excellent method repeatability and stability (Table S2 in see in SI). These results confirm that the developed HPLC-PDA method exhibits reliable performance for routine quantification of azithromycin, clarithromycin, and erythromycin in wastewater samples.

#### 3.3.6. Selectivity

The selectivity of the developed HPLC-PDA method was evaluated by analyzing blank water samples, standard solutions, and spiked samples containing azithromycin, clarithromycin, and erythromycin. As shown in Fig. 1, each analyte produced a well-defined peak with retention times of 3.76, 4.91, and 6.23 minutes, respectively. No interfering peaks were observed at these retention times in blank or spiked matrices, demonstrating baseline separation (*R*_s_ > 1.5) and excellent chromatographic resolution. The photodiode array spectra of the analyte peaks matched those of the corresponding standards, confirming the specificity of the method (see Fig. S1 in SI). These results indicate that the method is highly selective and suitable for the simultaneous quantification of macrolide antibiotics in environmental water samples.

#### 3.3.7. Robustness

The robustness of the developed HPLC method for simultaneous determination of erythromycin, clarithromycin, and azithromycin was evaluated by introducing small, deliberate variations in analytical parameters, including flow rate (±0.1 mL min^−1^), column temperature (±2 °C), and mobile phase pH (±0.2 units). The effect of these variations on retention time (*t*_R_), theoretical plate number (*N*), tailing factor (*T*_f_), resolution (*R*_s_), and %RSD of peak area was examined.

As summarized in Table S3 (in the SI), the *t*_R_ of the three antibiotics remained stable, with minor changes consistent with the applied variations. The *N* values remained high (erythromycin (E): 2080–2150; clarithromycin (C): 2490–2650; azithromycin (A): 2760–2890), indicating good column efficiency under all tested conditions. The *T*_f_ values for all analytes were within 1.05–1.10, confirming well-shaped, symmetric peaks. Furthermore, the *R*_s_ values between adjacent peaks (E-C and C-A) were always greater than 1.5, demonstrating adequate peak separation despite the variations.

The %RSD of peak areas for all analytes remained below 0.3%, confirming quantitative reproducibility under the tested conditions. These results indicate that the method is robust and reliable, and small variations in flow rate, column temperature, or mobile phase pH do not significantly affect the chromatographic performance or quantitative accuracy.

Overall, the method meets the ICH Q2 (R2) criteria for robustness, with all key chromatographic parameters remaining within acceptable limits under deliberate variations. This confirms that the developed HPLC method is suitable for routine analysis of erythromycin, clarithromycin, and azithromycin in wastewater.

### 3.4. Analysis of antibiotics in environmental samples

#### 3.4.1. Concentrations of macrolides in various water samples

**Fig. 8:**
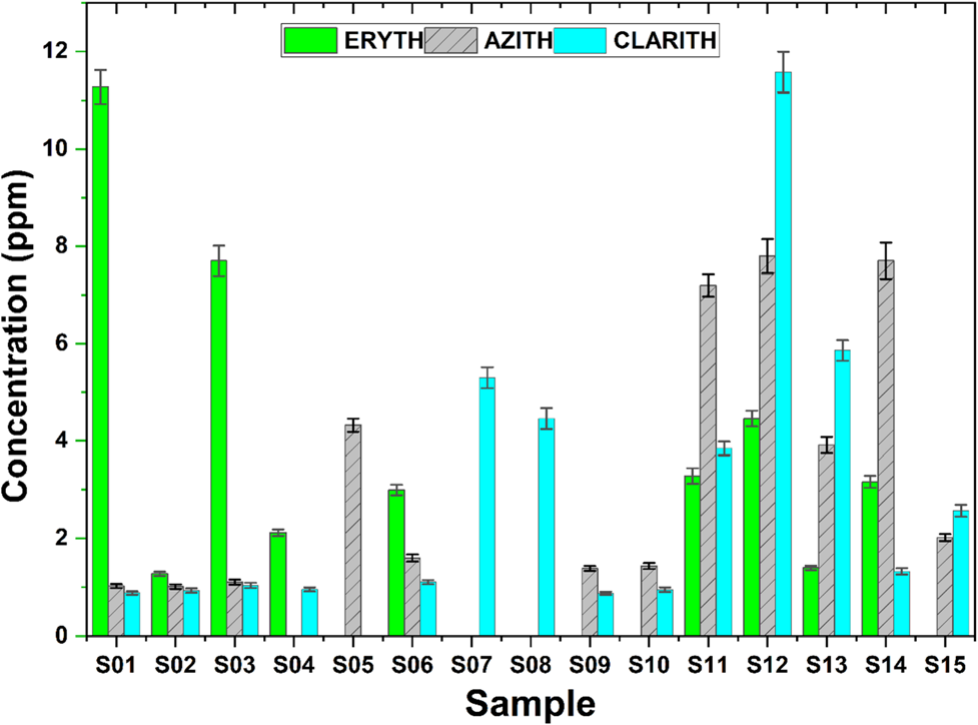
Measured concentrations of erythromycin (ERYTH), azithromycin (AZITH), and clarithromycin (CLARITH) in 15 wastewater samples (S01–S15). Bars represent the mean concentration (ppm), and error bars indicate ± standard deviation (*n* = 3).

Chromatographic measurements were performed on 15 real samples, with each sample analyzed in quintuplicate. Based on the recorded chromatograms and the established calibration curves, the concentrations of the target compounds in the real samples as well as the repeatability of the measurements were determined. The results are presented in Table S4 (see in SI), Table 10 and Fig. 8.

**Table 10:** Average Concentrations of erythromycin, clarithromycin, and azithromycin in real samples^a^

Real samples	Mean values of concentration (mg L^−1^)		
	ERYTH	CLARITH	AZITH
S1	11.273 ± 0.000	1.016 ± 0.003	1.023 ± 0.039
S2	1.275 ± 0.002	1.073 ± 0.003	1.011 ± 0.001
S3	7.701 ± 0.003	1.197 ± 0.003	1.103 ± 0.002
S4	2.118 ± 0.001	1.102 ± 0.000	ND
S5	ND	ND	4.321 ± 0.012
S6	2.994 ± 0.001	1.275 ± 0.010	1.598 ± 0.002
S7	ND	6.114 ± 0.013	< LOD
S8	ND	5.142 ± 0.009	< LOD
S9	< LOD	1.006 ± 0.008	1.383 ± 0.009
S10	ND	1.096 ± 0.007	1.435 ± 0.013
S11	3.284 ± 0.011	4.438 ± 0.010	7.196 ± 0.009
S12	4.458 ± 0.001	13.360 ± 0.009	7.795 ± 0.001
S13	1.398 ± 0.001	6.762 ± 0.011	3.914 ± 0.001
S14	3.164 ± 0.004	1.527 ± 0.007	7.702 ± 0.008
S15	< LOD	2.966 ± 0.000	2.020 ± 0.011

a ND: not detected (no peak above baseline noise), < LOD: below the limit of detection. Values are given as mean ± SD (*n* = 5).

From the analysis of 15 wastewater samples collected from hospitals and livestock farms in two provinces (Cao Bang and Ha Nam), it was observed that all samples contained at least one of the investigated antibiotics, with eight samples simultaneously containing all three compounds. Erythromycin was the most frequently detected, with concentrations ranging from 1.275 mg L^−1^ (S2) to 11.273 mg L^−1^ (S1). Clarithromycin was found in 12 of 15 samples, with the highest concentration observed in sample S12 (13.360 ppm) and the lowest in sample S9 (1.006 mg L^−1^). Azithromycin was detected in 9 of 15 samples, with concentrations ranging from 1.011 ppm (S2) to 7.795 mg L^−1^ (S12).

Overall, the concentrations of the target antibiotics were higher in hospital wastewater compared to livestock wastewater, with hospital samples from Ha Nam showing higher levels than those from Cao Bang province. This difference may be attributed to the larger patient population and consequently higher antibiotic usage in hospitals (Ha Nam province), as well as differences in hospital departments, treatment regimens, and wastewater management practices (see Fig. 8). In contrast, antibiotic concentrations in aquaculture and poultry farming areas were generally lower and varied across locations, reflecting more limited or inconsistent antibiotic use.

#### 3.4.2. Spatial or temporal trends observed

The spatial distribution of erythromycin, clarithromycin, and azithromycin varied markedly across the two provinces and between sample types. Overall, hospital wastewater samples exhibited substantially higher concentrations of the target macrolides compared to aquaculture and livestock wastewater. For instance, the highest levels were detected in Ha Nam hospitals (S11 – S12), where clarithromycin and azithromycin reached 13.36 mg L^−1^ and 7.80 mg L^−1^, respectively. In contrast, aquaculture ponds in both provinces (*e.g.*, S4, S5, S7, S8) generally showed either non-detectable levels or much lower concentrations, with only sporadic detections of single antibiotics. This clear separation indicates that hospital effluents are dominant point sources, while aquaculture contributes less consistently and at lower magnitudes.

At the provincial level, samples from Ha Nam contained higher antibiotic loads than those from Cao Bang, particularly for clarithromycin and azithromycin. This may reflect the larger patient population and higher antibiotic consumption in Ha Nam, combined with differences in wastewater management and treatment efficiency. Interestingly, erythromycin was frequently detected in Cao Bang hospitals (*e.g.*, 11.27 mg L^−1^ at S01), whereas clarithromycin dominated in Ha Nam hospitals (*e.g.*, 13.36 mg L^−1^ at S12), suggesting possible variation in prescription practices between provinces.

Regarding temporal trends, the present study was limited to a single sampling campaign conducted between August and September 2024, and therefore seasonal or longer-term variability could not be assessed. Nevertheless, the observed spatial contrasts highlight the importance of conducting systematic temporal monitoring, since antibiotic consumption in both human and veterinary sectors is likely to fluctuate with disease outbreaks, seasonal infection patterns, and agricultural cycles.

These findings underscore that spatial factors such as type of source (hospital *vs.* livestock), population density, and local wastewater treatment practices are the primary drivers of macrolide distribution in the studied regions. Future monitoring efforts that combine spatial and temporal data would enable a more comprehensive assessment of antibiotic release dynamics and their implications for antimicrobial resistance dissemination.

### 3.5. Comparison with existing methods

Different analytical methods for antibiotic determination each present distinct advantages and limitations in terms of sensitivity, operational simplicity, cost-effectiveness, and suitability for routine environmental monitoring.^13^Table 10 summarizes representative examples with quantitative performance metrics (LOD, LOQ, and validated range), together with a comparison to the present HPLC-PDA method (Table 11). The present HPLC-PDA method achieved limits of detection (LODs) of 5.0–6.0 µg L^−1^ and limits of quantification (LOQs) of 17–20 µg L^−1^ for azithromycin, clarithromycin, and erythromycin, with a validated linearity range of 2–15 µg mL^−1^. In contrast, LC-MS/MS methods reported by Kim *et al.* (2021) and Choi *et al.* (2020) achieved LODs of 0.2–2 µg L^−1^ but required expensive instrumentation and complex sample pretreatment. Earlier UV-Vis or HPLC-UV methods typically exhibited higher LODs (10–50 µg L^−1^) and narrower working ranges.

**Table 11:** Comparison of analytical methods for antibiotic determination and evaluation of the present HPLC-PDA method

Method	LOD (µg L^−1^ or ng L^−1^)	LOQ (µg L^−1^ or ng L^−1^)	Linear range	Ref.
LC-MS/MS/UPLC-MS/MS	0.04–1 ng L^−1^	0.1–3 ng L^−1^	0.001–1 µg L^−1^	45 and 46
HPLC-UV/DAD	0.2–2 µg L^−1^	0.6–6 µg L^−1^	0.1–10 mg L^−1^	13 and 47
Capillary electrophoresis (CE)	1–100 µg L^−1^	0.28–0.50 µg L^−1^	0.01–5 mg L^−1^	48 and 49
GC-MS	0.5–2 µg L^−1^	2–5 µg L^−1^	0.01–10 mg L^−1^	48 and 50
Electrochemical methods	0.01–0.1 µM (≈7–70 µg L^−1^)	5–56 ng L^−1^	Up to 0.5 mM (≈180 mg L^−1^)	51 and 52
This study (HPLC–PDA)	5–6 µg L^−1^	17–20 µg L^−1^	2.0–15.0 mg L^−1^	45 and 46

Although LC-MS/MS techniques offer the lowest detection limits at the ng L^−1^ level, their high instrumentation and maintenance costs restrict their routine use in many environmental laboratories, especially in developing countries. Other low-cost approaches such as UV/DAD, microbiological assays, capillary electrophoresis, or electrochemical methods provide simplicity and rapid analysis, but they often lack the selectivity, sensitivity, or reproducibility required for complex wastewater matrices. Within this context, the optimized HPLC-PDA method developed in this study provides a practical middle ground: it delivers sufficient sensitivity for macrolide concentrations commonly found in hospital and livestock effluents (µg L^−1^ to mg L^−1^), while maintaining operational simplicity, cost-efficiency, and strong analytical performance. Its validated linear range of 2.0–15.0 mg L^−1^, LODs of 5–6 µg L^−1^, and recoveries of 99.75–104.53% demonstrate that the method can reliably quantify macrolide antibiotics in complex matrices using standard HPLC systems, making it a robust and accessible tool for routine environmental monitoring.

### 3.6. Limitations and analytical scope of the developed method

Although It should be noted that the present study focuses on the development and validation of a robust HPLC-PDA method for the quantitative determination of azithromycin, clarithromycin, and erythromycin in wastewater. While forced degradation studies under acidic, basic, oxidative, thermal, and photolytic conditions are recommended by ICH Q1A (R2) and Q2 (R2) for comprehensive stability assessment, such experiments were considered beyond the scope of this work. The validation in this study was designed to address parameters critical for routine quality control, including accuracy, precision, linearity, LOD/LOQ, and robustness. Evaluation of degradation products and full stability profiling could be explored in future investigations.

Although the developed method demonstrated excellent precision, linearity (*R*^2^ ≥ 0.997), and accuracy (recoveries 99.75–104.53%), its instrumental detection limits (LOD = 5–6 µg L^−1^; LOQ = 17–20 µg L^−1^) are higher than the ng L^−1^ levels typically reported for surface waters. This limitation arises from the intrinsic sensitivity of UV-based detection compared with LC-MS/MS systems. Nevertheless, the current method was designed for the routine quantification of macrolides in untreated hospital and livestock wastewater, where the analyte concentrations are in the µg L^−1^ to mg L^−1^ range, well above the LOQ. The absence of detectable interferences and the strong reproducibility confirm its suitability for this purpose.

Our laboratory currently does not possess SPE facilities or LC-MS/MS instrumentation, and thus could not conduct further pre-concentration or ultra-trace validation. We explicitly acknowledge this limitation and note that future work will aim to collaborate with equipped laboratories to extend the method's applicability to ng L^−1^ detection. Despite these limitations, the present HPLC-PDA method remains a reliable, cost-effective, and accessible tool for continuous monitoring of macrolide antibiotics in wastewater treatment and pollution-source tracking.

## 4. Conclusion

A simple, cost-effective, and reliable HPLC-PDA method was successfully developed and validated for the simultaneous determination of azithromycin, clarithromycin, and erythromycin in environmental water samples. The method exhibited good linearity (2-15 µg mL^−1^), precision (%RSD < 2%), and accuracy (99.75-104.53%), with LODs and LOQs of 5–6 µg L^−1^ and 17–20 µg L^−1^, respectively. Application to real wastewater samples revealed that macrolide antibiotics were frequently detected in both hospital and livestock effluents, with concentrations reaching up to 13.36 mg L^−1^.

These findings highlight the persistent release of macrolide antibiotics into aquatic environments and the need for improved wastewater management practices to mitigate potential ecological risks. The developed HPLC-PDA method provides a practical and accessible analytical tool for routine monitoring of antibiotic contamination in regions lacking advanced instrumental facilities such as LC-MS/MS.

From a policy perspective, the method can support environmental surveillance programs and contribute to antibiotic stewardship initiatives by providing reliable data for regulatory agencies to evaluate the effectiveness of pollution control strategies. Future studies should focus on integrating the method with preconcentration or LC-MS/MS techniques to enable detection at trace (ng L^−1^) levels and to better assess long-term ecological impacts.

Overall, this study provides both a scientifically sound analytical framework and a practical contribution to environmental monitoring and management, supporting sustainable approaches to mitigate antibiotic pollution in aquatic ecosystems.

## Author contributions

Conceptualization: T. T. A. D. and T. X. V.; methodology: T. X. V., T. T. A. D., and T. T. T.; software: T. T. L. N. and T. M. V. N.; validation: T. X. V., T. T. A. D., and T. T. L. N.; data curation: T. T. A. D., T. T. T., and T. M. V. N.; writing original draft preparation: T. X. V. and T. T. A. D.; writing review and editing: T. T. T. and T. X. V.; visualization: T. T. L. N. and T. M. V. N. All authors have read and agreed to the published version of the manuscript.

## Conflicts of interest

The authors declare no conflicts of interest.

## Supplementary Material

RA-015-D5RA07172K-s001

## Data Availability

The data supporting this article have been included as part of the Supplementary Information (SI). The SI file provides detailed supporting data for the study, covering sampling sites (Table S1), method validation (Table S2, Table S3 and Fig. S1), and the quantification results of erythromycin (Eryth), clarithromycin (Clarith), and azithromycin (Azith) in real-world samples (Table S4) and are available from the corresponding author upon reasonable request. See DOI: https://doi.org/10.1039/d5ra07172k.
