# Supplementary material for: Development of an HPLC method for the simultaneous determination of azithromycin, clarithromycin, and erythromycin in wastewater
Source: RSC Adv. 2025 Dec 5;15(56):48197–215. doi: 10.1039/d5ra07172k (PMC12679586; doi:10.1039/d5ra07172k)
Supplement: RA-015-D5RA07172K-s001 [file RA-015-D5RA07172K-s001.pdf]

Supplementary Information (SI) for RSC Advances.
This journal is © The Royal Society of Chemistry 2025

Supplementary Information (SI)

Table S1. Sampling sites and times

Sample ID	Sampling site	Coordinates	Sampling time
S1	Obstetrics Department, Trung Khanh General Hospital<br>Address: Group 9, Trung Khanh Town, Cao Bang	22°50'19"N<br>106°31'37"E	23/08/2024, 07:00–09:00
S2	Pediatrics Department, Trung Khanh General Hospital<br>Address: Group 9, Trung Khanh Town, Cao Bang	22°50'19"N<br>106°31'35"E	
S3	Tra Linh General Hospital<br>Address: Cao Chuong, Trung Khanh, Cao Bang	22°48'26"N<br>106°19'51"E	
S4	Fish pond<br>Address: Po Tau, Chi Vien, Trung Khanh, Cao Bang	22°49'04"N<br>106°37'07"E	
S5	Fish pond<br>Address: Ban Da, Trung Khanh, Cao Bang	22°49'45"N<br>106°32'17"E	
S6	Quang Uyen General Hospital<br>Address: Hoa Nam Street, Quang Uyen Town, Quang Hoa,	22°41'19"N<br>106°26'44"E	

	Cao Bang		
S7	Fish pond<br>Address: Bang Hamlet, Duc Ly Commune, Ly Nhan District, Ha Nam	20°33'50"N<br>106°03'40"E	06/09/2024, 07:00–09:00
S8	Fish pond<br>Address: Duc Ly Commune, Ly Nhan District, Ha Nam	20°33'47"N<br>106°03'36"E	
S9	Poultry pond<br>Address: Do Hamlet, Bac Ly, Ly Nhan, Ha Nam	20°33'42"N<br>106°04'35"E	
S10	Poultry pond<br>Address: Yen Trach, Bac Ly, Ly Nhan, Ha Nam	20°33'34"N<br>106°04'24"E	
S11	Ly Nhan District General Hospital, Ha Nam<br>Address: Vinh Tru Town, Ly Nhan, Ha Nam	20°34'06"N<br>106°01'48"E	
S12	Ha Nam Provincial General Hospital<br>Address: Phu Ly City, Ha Nam	20°32'27"N<br>105°54'59"E	
S13	Pediatrics Department, Cao Bang Provincial General Hospital<br>Address: Km2,	22°39'11"N<br>106°16'11"E	11/09/2024, 07:00–09:00

	Dong Khe Road, Tan Giang Ward, Cao Bang City, Cao Bang		
S14	Endocrinology Department, Cao Bang Provincial General Hospital Address: Song Bang Ward, Cao Bang City	22°39'26"N 106°15'54"E	
S15	Cao Bang Traditional Medicine Hospital Address: Tan Giang Ward, Cao Bang City	22°39'23"N 106°15'46"E	

Table S2. Intra-day and Inter-day Repeatability (Single Concentration)

Analyte	Concentration (ppm)	Intra-day %RSD (n = 5)	Inter-day %RSD (n = 5)
Erythromycin	5	1.2	1.5
Clarithromycin	7	1.1	1.3
Azithromycin	10	1.3	1.4

Table S3. Effect of Deliberate Variations on Chromatographic Performance

Condition	Erythromycin [tR / N]	Clarithromycin [tR / N]	Azithromycin [tR / N]	Tf (E / C / A)	Rs (E–C / C–A)	%RSD Peak Area (E / C / A)
**Nominal (Flow = 0.8 mL/min; pH = 6.0; Temp = 35°C)**	[2.77 / 2120]	[3.34 / 2570]	[4.119 / 2840]	1.06 / 1.07 / 1.09	2.05 / 2.20	0.22 / 0.19 / 0.27
Flow = nominal + 0.1 mL/min	[2.76 / 2110]	[3.31 / 2550]	[4.118 / 2810]	1.05 / 1.06 / 1.08	2.00 / 2.15	0.22 / 0.19 / 0.27
Flow = nominal − 0.1 mL/min	[2.78 / 2130]	[3.36 / 2600]	[4.120 / 2890]	1.07 / 1.08 / 1.10	2.08 / 2.25	0.22 / 0.19 / 0.27
Column temp = nominal + 2 °C	[2.76 / 2100]	[3.33 / 2560]	[4.117 / 2760]	1.05 / 1.06 / 1.07	2.02 / 2.18	0.22 / 0.19 / 0.27
Column temp = nominal − 2 °C	[2.78 / 2140]	[3.35 / 2620]	[4.121 / 2840]	1.07 / 1.08 / 1.09	2.06 / 2.22	0.22 / 0.19 / 0.27
Mobile phase pH = nominal + 0.2	[2.79 / 2150]	[3.37 / 2650]	[4.119 / 2840]	1.08 / 1.09 / 1.10	2.10 / 2.28	0.23 / 0.19 / 0.27
Mobile phase pH = nominal − 0.2	[2.75 / 2080]	[3.31 / 2490]	[4.117 / 2760]	1.05 / 1.06 / 1.07	1.98 / 2.12	0.21 0.19 / 0.26

Note: E = erythromycin, A = azithromycin, C = clarithromycin (or your analyte names E, A, C as used in manuscript); %RSD (Area) calculated from 6 replicate injections under each condition; Rs = resolution; Tf = tailing factor; N = theoretical plate.

Table S4. Repeatability of Erythromycin, Clarithromycin, and Azithromycin Quantification in Real Samples (n = 5)

**Sample**	**Number of injection**	**ERYTH**		**CLARITH**		**AZITH**	
		Peak area (mAu)	Concentration (ppm)	Peak area (mAu)	Concentration (ppm)	Peak area (mAu)	Concentration (ppm)
**S1**	1	13421	11.273	802	1.014	787	1.007
	2	13421	11.273	804	1.016	787	1.007
	3	13421	11.273	805	1.017	789	1.008
	4	13421	11.273	809	1.020	892	1.093
	5	13421	11.273	801	1.013	781	1.002
	**SD**	0		3.114		47.5	
	**RSD(%)**	0		0.387 (%)		5.884 (%)	
**S2**	1	1097	1.277	879	1.077	791	1.010
	2	1097	1.277	871	1.070	793	1.012
	3	1092	1.273	875	1.073	791	1.010
	4	1093	1.274	873	1.072	792	1.011
	5	1095	1.276	873	1.072	794	1.012
	**SD**	**2.28**		3.033		1.304	
	**RSD(%)**	0.208 (%)		0.347 (%)		0.165 (%)	
**S3**	1	9019	7.703	1025	1.195	908	1.106
	2	9012	7.697	1023	1.193	904	1.103
	3	9013	7.698	1034	1.202	904	1.103
	4	9019	7.703	1029	1.198	903	1.102
	5	9019	7.703	1029	1.198	902	1.102
	**SD**	3.58		4.243		2.28	
	**RSD(%)**	0.04 (%)		0.413 (%)		0.252 (%)	
S4	1	2135	2.119	910	1.102	-	-
	2	2132	2.117	910	1.102	-	-
	3	2131	2.116	910	1.102	-	-
	4	2133	2.117	910	1.102	-	-
	5	2135	2.119	910	1.102	-	-
	**SD**	1.789		0		-	
	**RSD(%)**	0.084 (%)		0		-	
**S5**	1	-	-	-	-	4808	4.323
	2	-	-	-	-	4801	4.317
	3	-	-	-	-	4807	4.322
	4	-	-	-	-	4806	4.321

Sample	Number of injection	ERYTH		CLARITH		AZITH	
		Peak area (mAu)	Concentration (ppm)	Peak area (mAu)	Concentration (ppm)	Peak area (mAu)	Concentration (ppm)
	5	-	-	-	-	4808	4.323
	**SD**	-		-		2.915	
	**RSD(%)**	-		-		0.06 (%)	
**S6**	1	3215	2.995	1123	1.274	1507	1.600
	2	3214	2.994	1120	1.272	1501	1.595
	3	3213	2.993	1124	1.275	1503	1.597
	4	3214	2.994	1125	1.276	1505	1.599
	5	3213	2.993	1126	1.277	1505	1.599
	**SD**	0.837		2.302		2.28	
	**RSD(%)**	0.026 (%)		0.205 (%)		0.152 (%)	
**S7**	1	-	-	7091	6.111	-	-
	2	-	-	7099	6.118	-	-
	3	-	-	7096	6.115	-	-
	4	-	-	7095	6.115	-	-
	5	-	-	7093	6.113	-	-
	**SD**	-		3.033		-	
	**RSD(%)**	-		0.043 (%)		-	
**S8**	1	-	-	5897	5.144	-	-
	2	-	-	5893	5.140	-	-
	3	-	-	5894	5.141	-	-
	4	-	-	5895	5.142	-	-
	5	-	-	5897	5.144	-	-
	**SD**	-		1.789		-	
	**RSD(%)**	-		0.03 (%)		-	
**S9**	1	-	-	790	1.004	1246	1.385
	2	-	-	793	1.007	1241	1.381
	3	-	-	791	1.005	1241	1.381
	4	-	-	794	1.008	1243	1.383
	5	-	-	791	1.005	1245	1.384
	**SD**	-		1.643		2.28	
	**RSD(%)**	-		0.208 (%)		0.183 (%)	

Sample	Number of injection	ERYTH		CLARITH		AZITH	
		Peak area (mAu)	Concentration (ppm)	Peak area (mAu)	Concentration (ppm)	Peak area (mAu)	Concentration (ppm)
**S10**	1	-	-	902	1.095	1309	1.437
	2	-	-	903	1.096	1302	1.431
	3	-	-	904	1.097	1305	1.434
	4	-	-	905	1.098	1309	1.437
	5	-	-	902	1.095	1309	1.437
	**SD**	-		1.304		3.194	
	**RSD(%)**	-		0.144 (%)		0.244 (%)	
**S11**	1	3573	3.285	5027	4.439	8290	7.194
	2	3576	3.280	5023	4.435	8293	7.197
	3	3576	3.280	5025	4.437	8295	7.198
	4	3577	3.289	5027	4.439	8291	7.195
	5	3572	3.285	5029	4.440	8295	7.198
	**SD**	2.168		2.28		2.28	
	**RSD(%)**	0.06 (%)		0.045 (%)		0.183 (%)	
**S12**	1	5019	4.458	16034	13.360	9018	7.795
	2	5019	4.458	16038	13.363	9018	7.795
	3	5019	4.458	16034	13.360	9018	7.795
	4	5019	4.458	16035	13.360	9018	7.795
	5	5019	4.458	16032	13.358	9018	7.795
	**SD**	0		2.191		0	
	**RSD(%)**	0		0.014 (%)		0	
**S13**	1	1246	1.398	7890	6.759	4312	3.914
	2	1246	1.398	7892	6.761	4312	3.914
	3	1246	1.398	7896	6.764	4312	3.914
	4	1246	1.398	7895	6.763	4312	3.914
	5	1246	1.398	7896	6.764	4312	3.914
	**SD**	0		2.683		0	
	**RSD(%)**	0		0.034 (%)		0	
**S14**	1	3421	3.162	1432	1.525	8907	7.703
	2	3425	3.165	1436	1.528	8906	7.702
	3	3424	3.165	1435	1.527	8904	7.701
	4	3422	3.163	1437	1.529	8905	7.701
	5	3424	3.165	1435	1.527	8909	7.705
	**SD**	1.643		1.871		1.924	

Sample	Number of injection	ERYTH		CLARITH		AZITH	
		Peak area (mAu)	Concentration (ppm)	Peak area (mAu)	Concentration (ppm)	Peak area (mAu)	Concentration (ppm)
	**RSD(%)**	0.048 (%)		0.13 (%)		0.022 (%)	
**S15**	1	-	-	3210	2.966	2019	2.023
	2	-	-	3210	2.966	2016	2.020
	3	-	-	3210	2.966	2017	2.021
	4	-	-	3210	2.966	2012	2.017
	5	-	-	3210	2.966	2017	2.021
	**SD**	-		0		2.588	
	**RSD(%)**	-		0		0.128 (%)	

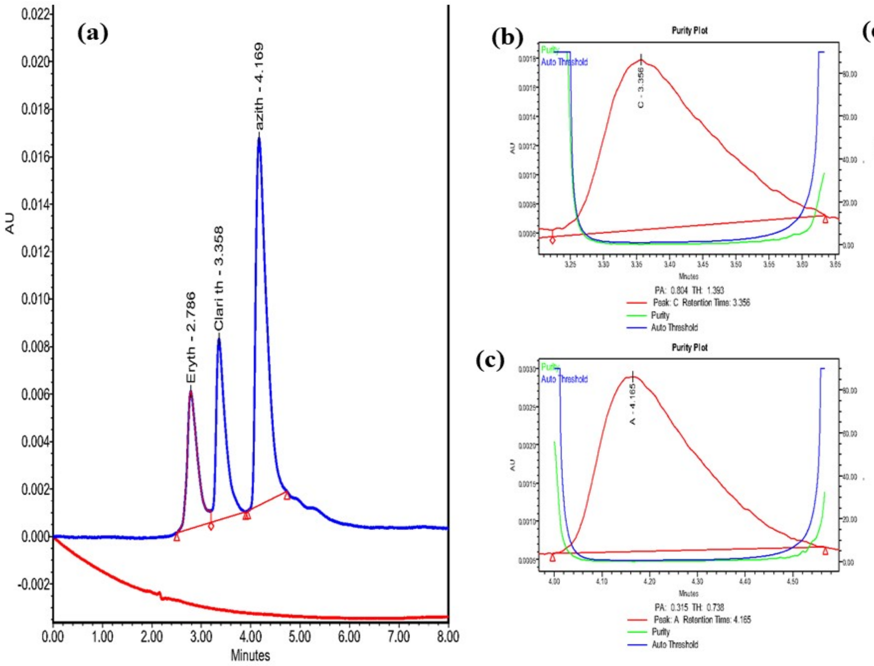

Figure S1. Selectivity (Specificity) assessment of the developed HPLC-PDA method. (**a**) Chromatogram of standard mixture of erythromycin (Eryth), clarithromycin (Clarith), and azithromycin (arith) at working concentrations, showing baseline separation with Rs > 1.5 between adjacent peaks (the blue line)and the chromatogram of blank (the red line). Purity plot of Peak purity of (**b**) clarithromycin (Clarith), (**c**) azithromycin (Arith) and (**d**) erythromycin (Eryth). Assessment using PDA detector confirmed spectral homogeneity of each analyte peak.
